# Eyebrow Lesion: An Unusual Suspect

**Published:** 2015-01-08

**Authors:** Andrew Marano, Alexis L. Parcells, Stephen R. Peters, Mark S. Granick

**Affiliations:** Division of Plastic Surgery, Department of Surgery, Rutgers New Jersey Medical School, Newark, NJ

**Keywords:** pleomorphic adenoma, lacrimal sac tumor, eyebrow lesion, lacrimal sac adenoma, cutaneous sweat gland adenoma

## DESCRIPTION

A 56-year-old man presents with a 1.5-cm lesion over his right eyebrow. The mass appears cystic and is affixed to the overlying skin. It has been present for 1 year and has not changed in size for several months. Patient denies pain or changes in vision.

## QUESTIONS

1. **What is the differential diagnosis for lesions of the eyebrow and upper eyelid?**
2. **What clinical presentation is associated with this lesion?**
3. **What is the recommended diagnostic and treatment paradigm for such lesions?**
4. **What is the risk of progression to malignancy?**

## DISCUSSION

**Figure 1:**
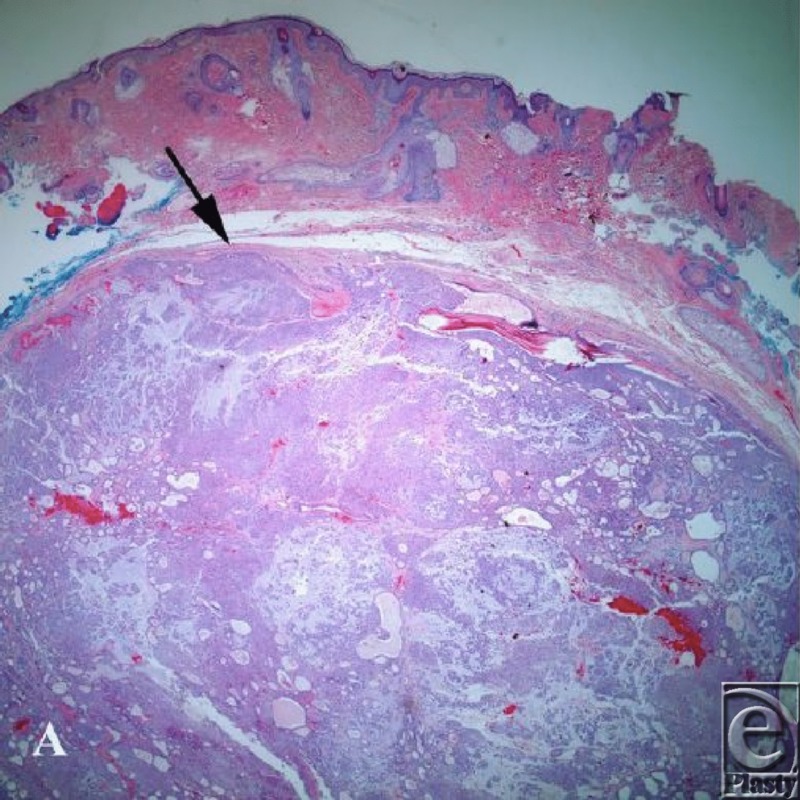
Hematoxylin and eosin stain demonstrates a well-circumscribed dermal nodule (arrow) composed of mixed solid cellular proliferation including microcysts, ducts, and areas of myxoid stroma.

**Figure 2:**
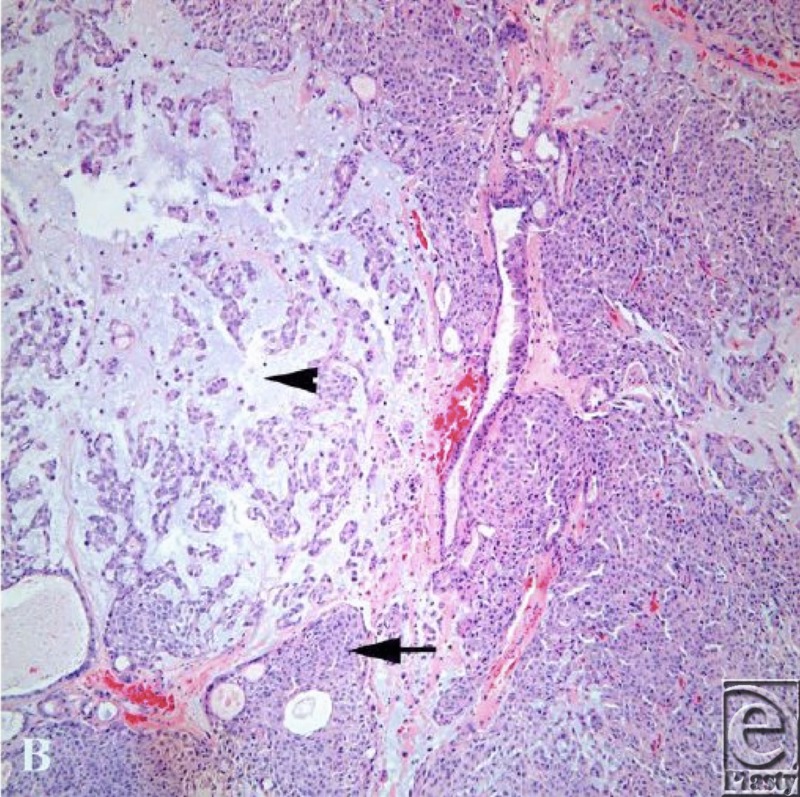
Hematoxylin and eosin stain demonstrates areas of cellular proliferation with squamoid differentiation, microcysts (arrow), and mixoid stroma (arrowhead). An area of duct differentiation is present centrally.

Common lesions of the upper eyelid and eyebrow include chalazion, inclusion cyst, pilomatricoma, dermoid cyst, sebaceous cyst, and basal cell carcinoma. These lesions are often excised and diagnosis is confirmed by gross and histologic examination. A pleomorphic adenoma is a benign biphasic tumor comprising epithelial and stromal components. Though commonly seen in salivary or parotid glands, a pleomorphic adenoma found in this region is rare and is usually derived from either a lacrimal or a cutaneous sweat gland. Histological findings for both derivations are very similar (Figs 1 and 2).^1^^-^2

When derived from a cutaneous sweat gland, the pleomorphic adenoma presents as a firm, nontender mass of the lid margin, central lid, or brow. It is typically a well-circumscribed nodule that may be mobile or fixed to the tarsus or overlying skin.^1^ Tumors derived from the lacrimal gland have a similar presentation. While tumors of the orbital lobe of lacrimal gland expand posteriorly within the orbit and may lead to proptosis, tumors of the palpebral lobe grow anteriorly and cause swelling of the upper lid.

**Figure 3:**
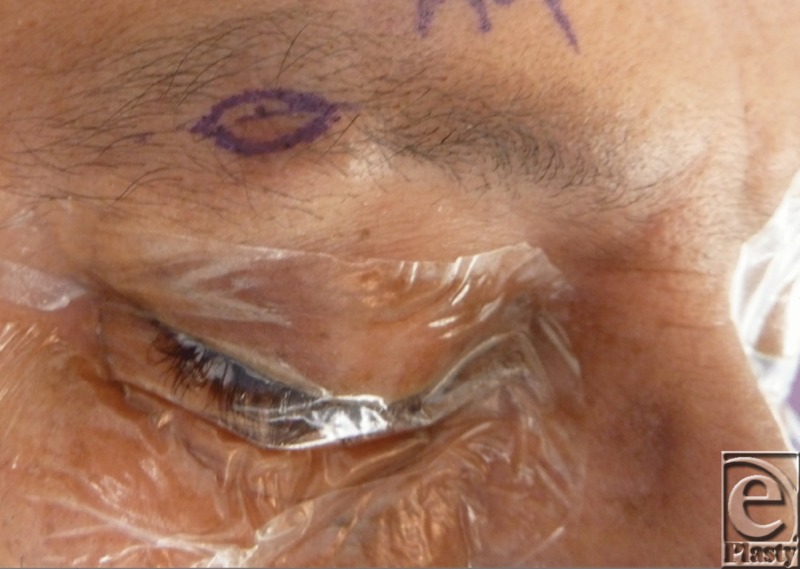
Preoperative marking of eyebrow lesion.

Diagnostic modalities for the lesions are controversial. A mass anterior to the orbital rim can often be appreciated on high-resolution computed tomography.^3^ Biopsy may theoretically disrupt the pseudocapsule and increases the risk of recurrence and progression. However, Lai et al investigated the outcomes of pleomorphic adenomas in the presence and absence of biopsy and concluded that all lesions should be biopsied for definitive preoperative diagnosis.^4^ The preferred management of pleomorphic adenomas is complete surgical excision. When derived from the palpebral lobe of the lacrimal gland, the most commonly documented method is removal via anterior or lateral orbitotomy (Fig 3).^3^^,^^5^ Mohs surgery has also proven effective in removal of these lesions.^6^

It is important that the tumor be excised with an intact pseudocapsule and clean margins as recurrence rates are as high as 30%, and progression to malignancy has been reported to be 30% after 15 years.^7^ In the case of malignancy, the recommended management is *en bloc* resection followed by radiotherapy or chemotherapy for small and circumscribed lesions, with consideration for orbital exenteration if mass has infiltrated beyond the capsule. Regular long-term follow-up is imperative, as the mean survival following diagnosis of carcinoma ex pleomorphic adenoma is 3 years.^8^ Our patient underwent complete excision of the lesion that measured approximately 1.5 × 1.0 × 0.8 cm^3^ and involved the subcutis.

Pleomorphic adenomas are benign glandular lesions. Though often found in parotid or salivary glands, they may rarely be seen originating from a lacrimal gland or cutaneous sweat gland. These lesions often present as well-circumscribed, firm, nontender masses. Complete excision with intact pseudocapsule and negative margins is important to prevent recurrence and malignant transformation.
